# Dysregulation of Group 3 Innate Lymphoid Cells in the Pathogenesis of Inflammatory Bowel Disease

**DOI:** 10.1007/s11882-016-0652-3

**Published:** 2016-09-19

**Authors:** Marianne Forkel, Jenny Mjösberg

**Affiliations:** Center for Infectious Medicine, Department of Medicine Huddinge, Karolinska Institutet, Stockholm, Sweden

**Keywords:** Innate lymphoid cells, ILC3, Inflammatory bowel disease, Intestinal homeostasis

## Abstract

**Purpose of Review:**

Here, we review recent literature indicating a role of innate lymphoid cells in human inflammatory bowel disease with a focus on the plastic population of ILC3.

**Recent Findings:**

Many studies suggest an involvement of ILC3 in human intestinal inflammation. ILC3 present the most abundant ILC subtype in the human intestine at steady state. In IBD, this composition is skewed towards ILCs showing an ILC1 phenotype and cytokine profile. This change is likely due to the microenvironment causing skewing of the functionally plastic ILC subsets. Interactions between ILCs and other cells are important to keep homeostasis and intestinal barrier integrity.

**Summary:**

The knowledge about the involvement of ILCs in IBD is rapidly increasing, and with the help of mouse models, new pathways and functions of ILCs are continuously unraveled. In the majority of human studies, a potential role for ILCs in Crohn’s disease is found. However, less data is available for a possible role in ulcerative colitis. Results from mice are obtained from diverse model systems, and more research in this field is needed to clarify and integrate the current knowledge in order to improve treatment strategies for IBD patients.

## Introduction

Inflammatory bowel disease (IBD) is an increasing medical problem with a strongly impaired quality of life for the patients. Until today, the causes and molecular mechanism of the disease are not clear and the available treatment regimens are unsatisfying. A role for adaptive immune cells in IBD has been established, and more recently, the innate immune system has attracted attention in the context of IBD. Many studies indicate a role for innate lymphoid cells (ILCs) in the pathogenesis of IBD. In this review, we aim to summarize the latest literature on this topic and to put the available studies into a broader context, providing an interpretation of the meaning for IBD disease pathogenesis.

## Classification of Human Innate Lymphoid Cells

Innate lymphoid cells (ILCs) are a recently discovered group of innate immune cells. They are detected in many organs (blood, tonsils, thymus, liver, gut, lung, skin, uterus, and bone marrow) [1] and are especially enriched in mucosal tissues of the human body. ILCs are a relatively rare cell type, comprising only about 0.1–13 % of CD45^+^ leukocytes depending on the organ [2]. Over the last years, ILCs have been shown to play crucial roles in the control of tissue homeostasis, as effector cells in the immune responses to infections and in inflammatory conditions [3].

ILCs develop from the common lymphoid progenitor (CLP) under the influence of IL-2Rγc signaling and expression of the inhibitor of DNA-binding 2 (Id2) [4]. Whereas the ontogeny of ILCs in mice is becoming increasingly understood [5], the exact developmental pathways of ILCs in humans are still unclear. Commonly, ILCs are defined by a lymphoid morphology, by the absence of markers for myeloid and dendritic cells (DCs) and, in contrast to T or B cells, by the lack of recombination activating gene (RAG)-dependent rearranged antigen receptors [6].

**Fig. 1:**
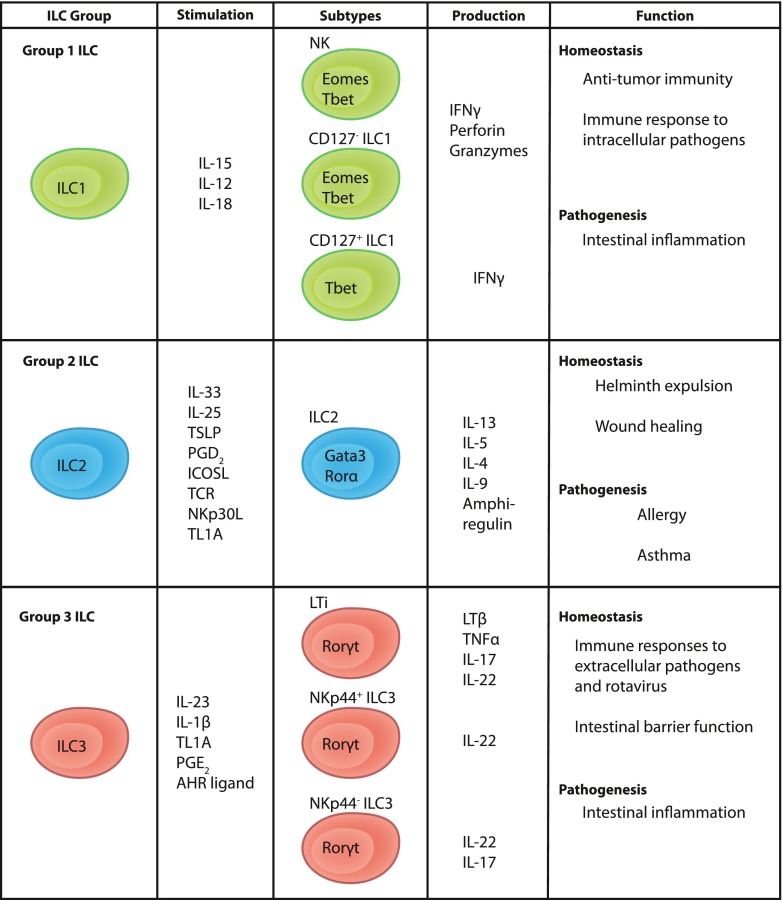
Classification of human ILCs. Human ILCs are divided into three broad subgroups called group 1, group 2, and group 3 ILCs based on the expression of transcription factors and cytokine production

Up until now, the family of ILCs has been divided into three subgroups, group 1, 2, and 3 ILCs (ILC1, ILC2, and ILC3), based on their transcription factor and cytokine production profile (Fig. 1). This classification is reflecting the classification of T helper cells in the adaptive arm of the immune system. Thus, ILCs are considered the innate counterparts of Th1, Th2, and Th17/22 cells. In contrast to T helper cells, ILCs respond rapidly to cytokine stimulation in the absence of specific antigens but once activated, they display similar effector functions as the respective groups of T helper cells [7].

Human ILC1 is a heterogeneous group comprised of NK cells, intraepithelial CD127^−^ ILC1 [8•], and CD127^+^ ILC1 [9••]. The former two subsets may be described as the innate counterparts of CD8^+^ T cells as they exert cytotoxicity and are hence not referred to as “helper” types of ILC1. In contrast, CD127^+^ ILC1 lack effector molecules associated with cytotoxicity, such as perforin and granzyme B, and are therefore more similar to CD4^+^ helper T cells. Hence, they are considered to be the innate equivalents of Th1 cells. Mouse studies have shown that intraepithelial ILC1 and conventional NK cells are different lineages, and that they are developmentally separated from helper ILCs [10••]. All ILC1 express the transcription factor T-bet whereas NK cells and intraepithelial ILC1 additionally express eomesodermin (Eomes). Commonly, ILC1 produce the signature cytokine IFN-γ following stimulation with IL-12 and IL-18 [11]. Together, the physiological role for ILC1 seems to be immune responses to intracellular pathogens and tumors.

ILC2 were first described in mice [12–14] and later in humans [15, 16] as cells dependent on the transcription factors Gata-binding protein 3 (GATA3) and retinoic acid receptor (RAR)-related orphan receptor α (RORα) for their development [17–19]. ILC2 display a type-2 cytokine profile. In response to stimulation with IL-33, IL-25, or TSLP [13, 19, 20], they produce mainly IL-13 and IL-5, but also IL-4, IL-6, IL-8, IL-9, GM-CSF and in mice, amphiregulin [14, 15, 19, 21]. In addition, they express the prostaglandin D_2_ (PGD_2_) receptor CRTH2 [16] and respond to PGD_2_ stimulation with the production of type 2 cytokines [22]. In mice, ILC2 were shown to play a role in helminth expulsion [13].

ILC3 are defined by the expression of the RAR-related orphan receptor-γt (RORγt) [23] and surface expression of CD117 (c-kit) [24]. They respond to stimulation with IL-23 and IL-1β with production of the Th17/Th22 cytokines IL-17A, IL-17F, and IL-22, either alone or in combinations [23, 24]. The group of human ILC3 includes fetal lymphoid tissue inducer (LTi) cells [25] and postnatal ILC3, which can be further divided according to the expression of the activating NK cell receptor NKp44 [26]. LTi cells are critically involved in the development of lymphoid organs during embryogenesis through secretion of lymphotoxin-β and TNF-α. In addition, LTi cells can express IL-17A and IL-22 [25]. Postnatally, NKp44^+^ ILC3 are producing IL-22 but little IL-17 whereas NKp44^−^ cells produce IL-17 but limited amounts of IL-22 [26]. ILC3 are generally involved in the immune response to extracellular pathogens [27].

All helper ILC subsets, i.e., with the exception of NK cells and intraepithelial ILC1, are phenotypically defined by expression of the surface proteins CD161 and CD127 (IL-7Rα) and dependence on IL-7 for their development [6].

## Inflammatory Bowel Disease

Inflammatory bowel disease (IBD) includes Crohn’s disease (CD) and ulcerative colitis (UC). IBD is a chronic inflammation of the gastrointestinal tract, commonly attributed to a dysregulated immune response against the intestinal bacteria in genetically susceptible individuals. In addition, environmental factors have been implicated in the onset of disease, since the disease emerges in developing nations with westernized lifestyle and industrialization. Some of these factors influencing the risk of IBD development either positively or negatively are smoking, appendectomy, diet, medications, and pollutants [28].

IBD severely reduces the quality of life for patients, with the most common symptoms being diarrhea, abdominal pain, rectal bleeding, weight loss, and fatigue. IBD patients are at increased risk of developing other chronic autoimmune disorders like psoriasis, ankylosing spondylitis, or primary sclerosing cholangitis. About 25 % of patients develop other extra-intestinal manifestations of IBD like arthritis, uveitis, or skin lesions [1, 29].

Up to now, the exact causes of IBD are not clear and there is no curative treatment available. Most conventional drug therapies, such as anti-inflammatory drugs, corticosteroids, or immuno-suppressants [30], aim at symptomatic treatment. Relatively recently, a group of new biological treatment specimen, including anti-TNFα antibodies, has dramatically improved the treatment options for IBD patients [31].

There are several differences observed in the type of inflammation present in UC or CD patients. Ulcerative colitis usually affects the distal colon with continuous inflammation, which is restricted to the mucosa. In contrast, Crohn’s disease can affect the entire gastrointestinal tract and shows a discontinuous and transmural inflammation [29]. In terms of the immune mechanisms involved, UC is thought to be driven by a type-2 response including increased levels of IL-13 and IL-5 in the intestine and high expression of the type-2 driving cytokine IL-33 and its receptor ST2 [32–34]. CD, on the other hand, is considered a type-1 driven inflammation, characterized by increased numbers of Th1 cells and high IFN-γ levels in response to IL-12 [32, 33, 35].

The microbiota plays a crucial role in disease pathogenesis. IBD patients present with dysbiosis, changes in microbiota, including a reduced diversity of *Firmicutes* and *Bacteroidetes* [36]. A recent study on treatment-naïve pediatric CD samples investigated the microbiotic changes in more detail and suggested sampling of the mucosal microbiota as a diagnostic tool for IBD [37]. In patients suffering from IBD, the intestinal epithelium shows an increased permeability, the “leaky-gut phenomenon”, which permits enhanced immune responses against the commensal microbiota [29].

Until today, it is in large parts still unclear which of the observed symptoms and immunological changes in IBD are cause or consequence of the disease and more research in this field is urgently needed.

## ILC in Gut Homeostasis and Inflammatory Bowel Disease

### ILC3 in Gut Homeostasis

In the healthy human intestine, all currently known ILC populations are present with ILC3 constituting the majority of ILCs in the lamina propria of the ileum [9••] whereas published data on the distribution of the ILC subsets in the healthy human colon is missing.

**Fig. 2:**
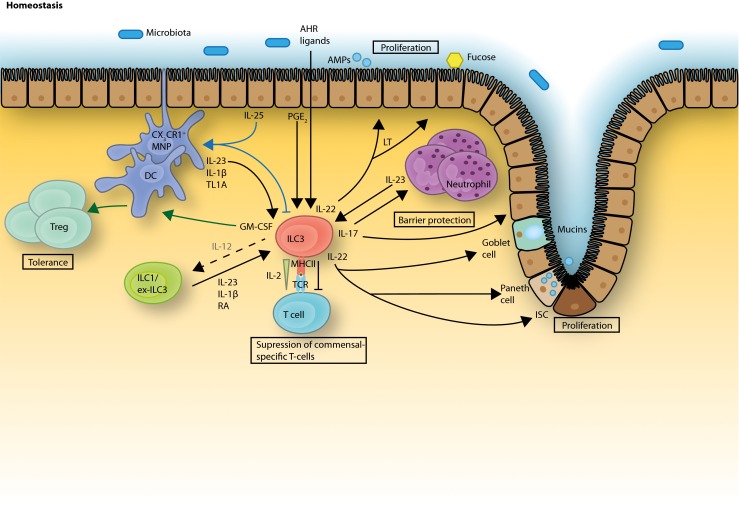
ILC3 functions in gut homeostasis. ILC3 are stimulated by IL-23, IL-1β, and TL1A, derived from MNPs and DCs to produce their signature cytokines IL-22 and IL-17 as well as GM-CSF. IL-22 promotes epithelial barrier integrity and proliferation, induces antimicrobial peptide and mucin production and, together with lymphotoxin, enhances epithelial fucosylation. IL-22 production from ILC3 can be also stimulated with epithelial cell-derived PGE_2_ whereas dietary AHR ligands help maintain ILC3 in the intestine. IL-17 induces the recruitment of neutrophils and also supports epithelial barrier protection. GM-CSF has been shown to be important in the induction of oral tolerance acting via DCs/MNPs. ILC3 were shown to inhibit commensal-specific T cells via the MHCII receptor together with a withdrawal of IL-2. Epithelial cell-derived IL-25 acts via MNPs/DCs to inhibit ILC3 functions. IL-23, IL-1β, and RA can induce plasticity of ILC1 and ex-ILC3 towards an ILC3 phenotype, whereas IL-12 can reverse this effect under inflammatory conditions

ILC3 are shown to be crucially involved in intestinal homeostasis (Fig. 2). Generally, ILC3 are known as producers of IL-22 and/or IL-17 in response to IL-23 and IL-1β. IL-22 acts on non-hematopoietic cells, such as epithelial cells, and through its heterodimeric receptor, consisting of IL-22R1 plus IL-10R2, and STAT-3 signaling induces mucosal wound healing responses and epithelial cell proliferation [38, 39]. In mice, it was further shown to mediate protection against intestinal pathogenic bacteria by inducing the production of a variety of antimicrobial peptides, such as RegIIIβ, RegIIIγ, S100A8, and S100A9, element-sequestering proteins, and mucins [40].

A second mechanism directly influencing the intestinal microbiota was recently described in mice. ILC3 are involved in the induction of Fut2 in intestinal epithelial cells, an enzyme which mediates epithelial cell fucosylation [41]. This induction required commensal-dependent IL-22 and commensal-independent lymphotoxin (LT) production. Epithelial fucose is catabolized by commensal bacteria and in this way supports homeostasis of the microbiota and protection from pathogenic bacteria.

Another beneficial effector function of ILC3-derived IL-22 was observed in a mouse model of graft-versus-host disease (GVHD) and tissue damage, where IL-22 acts on intestinal stem cells to preserve the epithelial barrier integrity [42••, 43]. IL-22 production by ILC3 is enhanced through activation of the transcription factor aryl hydrocarbon receptor (AHR). AHR is a ligand-activated transcription factor with many ligands, such as tryptophan metabolites, occurring in the diet and environment [44].

Another ILC3-derived cytokine, IL-17, is known to induce the release of chemokines and other chemoattractants from epithelial and endothelial cells, which enhance the inflammation response through recruitment of pro-inflammatory neutrophils [45]. However, protective effects of IL-17, for example, in IBD, are also reported. One of the main inducers of IL-17, IL-23, can be targeted by ustekinumab (anti-IL-12p40) for successful IBD treatment [46]. However, targeting IL-17 itself through secukinumab actually exacerbates IBD [47•]. This dichotomy was recently explained by the observation that IL-17 promotes epithelial barrier function in the absence of IL-23 [48, 49•]. Hence, IL-23 independent IL-17 production is protective, whereas IL-23 dependent IL-17 production might be deleterious, as suggested by the numerous mouse studies discussed in the next section.

How ILC3 are integrated into a complex network of intestinal immune cells is crucial for the understanding of their function, and therapeutic potential has only started to be unraveled. A major source of the ILC3-stimulating factors IL-23 or IL-1β in the human gut is CX_3_CR1^+^ mononuclear phagocytes (MNPs) or CD14^−^ dendritic cells [50••, 51]. Mononuclear phagocytes play an important role in the maintenance of gut homeostasis. They can directly sense commensal bacteria and subsequently stimulate ILC3 via IL-1β, IL-23, and TL1A to produce effector cytokines IL-22 and GM-CSF [51]. In a feedback loop, GM-CSF is a crucial regulator of mononuclear phagocytes, which induce intestinal Treg cells [52]. In this way, the crosstalk between mononuclear phagocytes and ILC3 is important in the induction of tolerance towards commensals and dietary antigens. In mice, it was shown that ILC3 responses are indirectly down-regulated via IL-25, which is secreted by microbiota-stimulated epithelial cells and likely acts via DCs, which in turn limits ILC3 activation through a so far unknown mechanism [53].

In addition to being regulated by immune cell subsets such as DCs, ILC3 has the capacity to regulate the activity of other immune cells via direct cell-cell interactions with important implications for gut homeostasis. ILC3 were shown to regulate adaptive T cell responses through the expression of MHCII molecules. So far, most data are based on mouse studies and the expression pattern of MHCII molecules significantly differs between mouse and human. However, it seems likely that ILC–T cell interactions are also taking place in the human setting, since MHCII (HLA-DR) expression was detected on human intestinal ILC3 [54••] and defines a transcriptionally and functionally distinct ILC3 subset in the human tonsil [55•]. In the mouse, ILC3 limited commensal bacteria-specific T cell responses through MHCII-dependent interactions with CD4^+^ T cells and this effect was independent of ILC3 effector cytokines. Later, it was shown that this intestinal T cell selection is achieved by antigen presentation together with IL-2 cytokine withdrawal mediated by MHCII^+^ ILC3 leading to programmed cell death of activated microbiota-reactive T cells [56•]. Furthermore, ILC3 were able to process and present antigen but lacked expression of the classical co-stimulatory molecules (CD40, CD80, CD86) [54••]. In contrast, it was shown that ILC3 isolated from mouse spleen express co-stimulatory molecules and induce T cell responses and proliferation [57]. These controversial observations might depend on the different microenvironments in different anatomical locations, which favor variable surface protein expression and diverse effector functions of ILC3. Nevertheless, the capacity of ILC3 to regulate T cell activity partly explains their dramatic importance, at least in certain mouse models, despite their relative rareness.

In summary, ILC3 deploy an array of cytokine-dependent and cell surface receptor-mediated mechanisms to exert homeostatic control of intestinal immunity.

### ILC3 in Inflammatory Bowel Disease

**Fig. 3:**
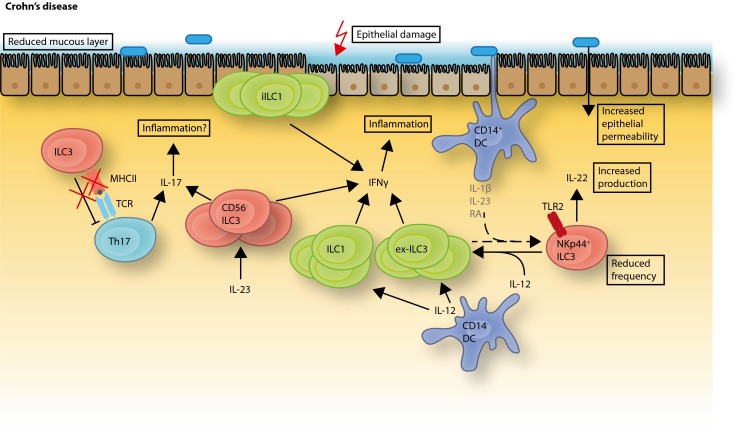
Changes of ILC frequencies and functions in intestinal inflammation. In the inflamed intestine of Crohn’s patients, several changes in the distribution of ILC frequencies have been observed. Homeostatic NKp44^+^ ILC3 are decreased whereas the frequencies of the IFN-γ-producing subsets of intraepithelial ILC1 and CD127^+^ ILC1 are increased. The IL-12 induced plasticity of NKp44^+^ ILC3 to ex-ILC3, resembling ILC1 is likely contributing to this increased frequency. Somewhat contradictory, NKp44^+^ ILC3 from the colon of UC and CD patients were shown to have a higher capacity for IL-22 production. An IL-17- and IFN-γ-producing CD56^−^ subset of ILC3 was found to be increased in the intestine of Crohn’s patients. In addition, the MHCII expression on intestinal ILC3 from pediatric Crohn’s patients was shown to be reduced and this correlated with an increased number of Th17 cells

Naturally, since a number of mouse models that mimic gut inflammation are available, a large amount of data indicating a role for ILCs in IBD is obtained from mouse studies. Nevertheless, many reports also indicate a role of ILCs in the promotion of IBD in humans (Fig. 3). However, the heterogeneity and plasticity within the group of ILC3, the different gating strategies used to identify ILC3, as well as the diversity of mouse models employed contribute to some confusion regarding the role for ILC3 in human gut inflammation.

Many genes related to ILC3 biology were identified in genome-wide association studies (GWAS) as risk loci for IBD. One key GWAS identified 163 risk loci for IBD, containing genes involved in autophagy, several genes of the IL-23/IL-17 pathway, mucins and *NOD2*, a gene important for intracellular pathogen sensing [58]. Since human ILCs express a large number of these GWAS genes [55•], it is possible that some IBD risk genes partly mediate their penetrance by modifying ILC functions.

Indeed, a number of studies reported changes in the ILC composition and function within the lamina propria of IBD patients connected to those risk genes. These findings in humans are supported by data from different mouse models of intestinal inflammation. Two studies identified a decrease of IL-22 producing ILC3 in the intestine of Crohn’s patients [9••, 59]. The protective effect of ILC3-derived IL-22 in gut inflammation was demonstrated in several mouse models. IL-23-induced IL-22 was shown to attenuate colon inflammation induced by dextran sodium sulfate (DSS) or *Citrobacter rodentium* infection [60] and lack of IL-22 producing ILCs increased susceptibility to *C. rodentium* [27, 61]. Furthermore, in the TCRα KO mouse model, representing a model of UC, IL-22 gene delivery improved colitis through mucin induction and goblet cell restitution [62]. However, in contrast to these protective and regenerative effects of ILC-22, Longman et al. found that colonic ILC3 from UC as well as CD patients showed an increased IL-22 production capacity [51] and a study using the anti-CD40 model of innate colitis found a pathogenic role for IL-22 [63]. Hence, ILC3-derived IL-22 has a complicated role in the intestine and cannot be easily designated as protective.

Takayama et al. and Bernink et al. showed a decrease of IL-22 producing NKp44^+^ ILC3 in the lamina propria of CD patients [9••, 59] but not UC patients [59]. One of the groups showed that the decrease of NKp44^+^ ILC3 was accompanied by a reciprocal increase of an IL-23 responsive IFN-γ-producing cell population which showed an NK cell phenotype (CD3^−^ CD56^+^ NKp44^−^NKp46^+^ CD127^−^ RORgt^−^) [59]. Bernink et al., on the other hand, identified a population of CD127^+^ ILC1 in humans that was found to be accumulated in the lamina propria of CD patients at the expense of NKp44^+^ ILC3. These cells produced IFN-γ under stimulation with IL-12 and were unresponsive to IL-23 or IL-1β [9••]. Another ILC1 subset, called intraepithelial ILC1, which resides in the intestinal epithelium and produces large amounts of IFN-γ upon IL-12 stimulation, was also amplified in the small intestine of CD patients [8•]. These intraepithelial ILC1 were characterized by expression of CD103, NKp44, granzymes, and perforin and they expressed the transcription factors Tbet and Eomes [8•]. Hence, several studies point to an accumulation of, and a pathogenic role for, IFN-γ producing ILCs in human gut inflammation, as shown in mice [64]. Indeed, IFN-γ is known to promote gut inflammation by reducing tight junction integrity, enhancing leukocyte recruitment, and stimulating phagocytes [65].

Not only an increase of IFN-γ in the intestine of IBD patients was observed but also a pro-inflammatory role for IL-17 was suggested. An increased frequency of IL-23 responsive IL-17- and IFN-γ-producing CD56^−^ ILC3 in the intestine of CD patients, but not in UC patients, was detected [66], and a similar subset was found at elevated numbers in a mouse model of *Helicobacter hepaticus*-induced colitis [64]. In addition, a mouse model of UC in Rag2^−/−^ and Tbet^−/−^ mice (TRUC mice) suggested a central role for IL-23-induced IL-17 in chronic intestinal inflammation [67]. However, as mentioned above, IL-23 and IL-17 seem to have opposed, yet linked, roles where anti-IL-23 treatment improves both IBD and experimental colitis [46, 47•, 49•, 68], whereas anti-IL-17 treatment worsens IBD [47•].

The skewed frequencies of ILCs in the intestine of IBD patients could either be explained by specific recruitment of cells to the intestine or by plasticity between the different ILC subsets, induced by the inflammatory response. Indeed, a high degree of plasticity among ILC subsets started to be recently unraveled. Human ILC3 were shown to differentiate into IFN-γ-producing ILC1 under IL-12 stimulation. IL-12 induced down-regulation of RORγt, up-regulation of Tbet and the cells lost the ability to produce IL-22. These ILC1-like cells are termed “ex-ILC3” [9••]. A similar conversion of RORγt^+^ ILC3 to IFN-γ-producing RORγt^−^ ILCs under IL-12 stimulation was reported in mice [69]. Recently, IL-12 was also shown to play a role in ILC2 plasticity. An increased frequency of ILC2 in intestinal samples of Crohn’s patients was found, and those cells had the ability to co-produce IL-13 and IFN-γ [70•]. In vitro ILC2 could be induced to express IFN-γ and Tbet under the influence of IL-12. If this mechanism could play a role in the pathogenesis of UC is to date unclear, and other reports studying the involvement of ILC2 in human IBD are eagerly anticipated. IL-12 is a pro-inflammatory cytokine produced mainly by antigen presenting cells in response to bacterial products [71] and is present at high levels in the inflamed gut mucosa. Clinical trials with a monoclonal antibody against the shared p40 subunit of IL-12 and IL-23 could induce remission in Crohn’s disease [72], but it is still unclear through which molecular mechanism improvement is achieved.

The conversion of ILC3 into ILC1 was recently shown to be reversible. CD127^+^ ILC1 could differentiate into RORγt^+^ ILC3 under stimulation with IL-2, IL-23, and IL-1β, and this was further enhanced by retinoic acid (RA) [50••]. This reversible plasticity is an elegant mechanism through which ILCs can be instructed to adapt to the current needs in a certain microenvironment without the recruitment of cells from the circulation.

Not only a skewed profile of ILC3 effector cytokines but also altered interactions of ILC3 within the intestinal immune cell network is suggested to play a role in intestinal inflammation. ILC3 and T cell interaction seems to have a crucial function in keeping gut homeostasis. Mice with a deletion of MHCII in the ILC3 developed spontaneous intestinal inflammation in the presence of commensal bacteria with increased frequencies of pro-inflammatory (IFN-γ ^+^, IL17A^+^, TNFα^+^) CD4^+^ T cells in the colon [54••]. In addition, it was found that MHCII expression on colonic ILC3 from pediatric IBD patients is reduced together with an increased frequency of Th17 cells in the colon [56•], which suggests a protective regulatory effect of MHCII expressing ILC3 in IBD.

As described above, the interaction of mononuclear phagocytes and ILC3 is important in the induction of intestinal tolerance towards commensals and dietary antigens. In the context of IBD, some studies indicate that this crosstalk could be disturbed in CD patients [52, 73, 74].

Furthermore, ILC3-derived IL-22 was shown to prevent the dissemination of lymphoid-resident bacteria, specifically *Alcaligenes*, in mice [75]. Crohn’s disease is associated with specific immune responses against *Alcaligenes*, which suggests a dysregulation of this mechanism in human IBD [75].

It is known that patients with inflammatory bowel disease have an increased risk of developing colorectal cancer. As described above, the IL-23 pathway is involved in intestinal inflammation in several mouse models and IL-22 has a protective effect in some models of intestinal inflammation. On the other hand, in the development of cancer, IL-22 can have a pro-tumorigenic effect. One study reported ILC3- and IL-22-dependent progression of colitis-associated colorectal cancer in a mouse model [76•], which indicates the possibility of an involvement of ILC3 in human colorectal cancer. These opposing protective and pathogenic effects of IL-22 indicate that a tight regulation of this cytokine is necessary to maintain homeostasis. An important regulator of IL-22 is the DC-derived IL-22 binding protein (IL-22BP). Mice lacking IL-22BP showed increased incidence of tumors in an IBD-associated colorectal cancer model [77].

## Conclusions

Important advances in our knowledge about immunological mechanisms in intestinal inflammation have been made, even if the initial trigger for the development of IBD is still unidentified. It is becoming increasingly clear though, that dysregulated ILCs are involved in the pathogenesis of IBD, especially through the usage of mouse models of intestinal inflammation. Different subsets of ILCs are expanded in the disease states compared to homeostasis, and more and more interactions of ILCs with other immune cells are being discovered. However, many questions concerning which of the observed alterations in ILC composition and function are causes or consequences of the inflammation are still unanswered.

Most research on humans investigating the role of ILCs in IBD focused so far on Crohn’s disease, and not much data is available for ulcerative colitis. Since UC is thought of as a type 2 mediated inflammation, an interesting question is if any changes in the ILC2 compartment and their function can be observed in the disease state.

Integrating and expanding the knowledge obtained from mouse models and confirming which role ILCs play in human IBD will lead to a better understanding of intestinal immune networks and will hopefully pave the way for better and finally curative treatment of IBD.
